# Wireless Positioning in IoT: A Look at Current and Future Trends

**DOI:** 10.3390/s18082470

**Published:** 2018-07-30

**Authors:** Pedro Figueiredo e Silva, Ville Kaseva, Elena Simona Lohan

**Affiliations:** 1 Laboratory of Electronics and Communications Engineering, Tampere University of Technology, Korkeakoulunkatu 3, Tampere 33720, Finland; elena-simona.lohan@tut.fi; 2 Wirepas, Visiokatu 4, Tampere 33720, Finland; ville.kaseva@wirepas.com

**Keywords:** Internet of Things (IoT), wireless positioning, indoor location

## Abstract

Connectivity solutions for the Internet of Things (IoT) aim to support the needs imposed by several applications or use cases across multiple sectors, such as logistics, agriculture, asset management, or smart lighting. Each of these applications has its own challenges to solve, such as dealing with large or massive networks, low and ultra-low latency requirements, long battery life requirements (i.e., more than ten years operation on battery), continuously monitoring of the location of certain nodes, security, and authentication. Hence, a part of picking a connectivity solution for a certain application depends on how well its features solve the specific needs of the end application. One key feature that we see as a need for future IoT networks is the ability to provide location-based information for large-scale IoT applications. The goal of this paper is to highlight the importance of positioning features for IoT applications and to provide means of comparing and evaluating different connectivity protocols in terms of their positioning capabilities. Our compact and unified analysis ends with several case studies, both simulation-based and measurement-based, which show that high positioning accuracy on low-cost low-power devices is feasible if one designs the system properly.

## 1. Introduction

Nowadays, the amount of connected wireless devices is growing, e.g., smart watches, smart light bulbs, smart toothbrushes, smart coffee mugs, etc. The trend in the information technology industry is towards connecting and extracting analytics from a variety of inter-connected wireless devices.

While many IoT applications have so far focused on the consumer realm, more and more industrial applications are also appearing, such as utilities measurement (e.g., water, electricity and gas), industrial lighting, logistics and smart agriculture. Enabling such industrial applications means that IoT networks need to support large amounts of devices, multiple years of operation on battery, different latency requirements and low costs per unit.

We believe that, on top of the communications and reliability requirements of a wireless link, many IoT applications will require or benefit from knowing the location of certain devices. Such location information will be needed seamlessly, both indoors and outdoors, and without the battery-draining Global Navigation Satellite Systems (GNSS) chipsets. The need for localization and tracking appears not only from the network management point of view, but also from a business perspective, driving new business models and new business avenues.

Nevertheless, enabling or creating a positioning system with an IoT network is not a trivial task. The reason behind this is that industrial applications seek a low per unit cost of their IoT devices, which results in devices with very limited hardware components, such as CPU, memory and battery. The limited hardware has an impact on the number of devices that a single device can serve and how fast it can process network and application requests. However, while CPU and memory will have an important impact on the scale of the network, the biggest challenge for enabling a positioning system lies on the proper management of the devices’ radio.

The need for proper radio management becomes evident as there are devices with known coordinates which will broadcast specific payloads on a regular basis and other devices whose locations are to be determined, which will need to scan the spectrum frequently. Hence, too frequent broadcasting will lead to spectrum congestion and increased packet collision, whereas frequent scanning leads to high battery consumption, which is particularly problematic for battery-operated devices.

Overall, the biggest challenge to tackle for an IoT positioning network is to balance the power consumption against the performance of the system. A very reactive system will have to rely on frequent scanning and broadcasting of its members, which means that devices will need to draw large quantities of power. A low reactive system will draw less power with devices scanning very seldom.

The goal of this paper is to provide an insight on positioning capabilities of the current IoT technologies and other relevant IoT-enabling wireless technologies, such as WiFi. The paper starts by classifying three domains of positioning and discussing the main shortcomings of each of these domains for IoT devices. It then classifies the different IoT solutions according to six classification criteria and it provides a discussion on the main system parameters relevant to positioning and tracking purposes. This discussion acts as a basis for comparison between the different IoT wireless solutions. To further complement this discussion, we present positioning results based on simulation-based scenarios and field experiments with a platform built on top of the Wirepas Mesh connectivity solution. In the end, we provide a short summary and conclusions of our findings.

## 2. Related Work

At this moment, to the best of the authors’ best knowledge, there are no comprehensive comparisons in the literature between different IoT protocols in terms of their positioning capabilities. There are, however, other studies that compare specific IoT technologies and which look at IoT from the communications point of view, as well as studies focusing on positioning with a particular technology, such as narrow-band IoT (NB-IoT) or BLE. In this section, we highlight the related work from literature studies.

A survey of localization methods for 5G, having a short section also on IoT positioning has been recently published as a white paper in COST action CA15104 [1]. It has also been emphasized in this paper that localization will become a key component of future 5G systems and it has been pointed out that accurate future localization solutions in 5G should exploit the multipath and non-line-of-sight information and should put more emphasis on heterogeneous data fusion mechanisms. However, such advanced solutions would also increase the power consumption on the devices and are not well-suited for the majority of IoT systems. By distinction with the work in [1], our paper focuses mostly on low-cost low-power consumption IoT solutions.

The authors in [2] focus on the Long-Term Evolution (LTE) Machine type communications (LTE-M) and Narrow Band Internet of Things (NB-IoT) protocols and their positioning capabilities. It was shown in [2] that at 46 dBm power of the transmit AN, positioning accuracy goes to around 10 m and that NB-IoT protocol supports better positioning accuracy than LTE-M protocol. A related study can be found in [3]. The focus in [3] is on indoor localization via improved received signal strength (RSS) fingerprinting in generic IoT devices. The results are based on 802.11n/b/g signals where location errors below 5 m are achieved in more than 50% of the studied cases.

In [4], the authors investigate a time-domain based positioning with additional frequency hopping for the NB-IoT system. The obtained positioning accuracy is down to 30–50 m under strong signal-to-noise ratio conditions, and it deteriorates quickly for medium and low signal-to-noise ratios.

A study complementary to our work is found in [5], where IoT positioning is looked at from the perspective of security, privacy and robustness of the localization technology. No positioning results were reported in the study. Another complementary study is found in [6] in which the authors focused on existing and emerging software and hardware platforms for IoT applications, but positioning was not part of that study. IoT positioning has recently been considered in [7] from the point of view of spoofing resistance in time of arrival (TOA) ultra-wideband (UWB) for IoT systems.

Other complementary comprehensive studies, focusing solely on the communication aspects of IoT, are found for example in [8,9].

## 3. Designing an IoT Positioning System

In its essence, a positioning system translates a set of measurements from well-known reference points into a coordinate pair. The reference points, known as anchors in localization terminology or Access Nodes (AN) in IoT terminology, act as a means for the device of interests, a mobile or an IoT tag, to be in a local or global reference frame. Depending on who makes the measurements, the positioning is considered to be network centric (i.e., when the anchors make the positioning-related measurements) or device centric (i.e., when the IoT end nodes or tags perform the positioning-related measurements).

These two types of positioning have very different implications on security and privacy, which should always be carefully considered regarding the final application. For example, privacy-preserving positioning solutions are easier to be achieved in a device-centric approach than in a network-centric approach as the device would not need to disclose its position to the network.

### 3.1. Positioning Domains

In terms of measurements, there are multiple domains from which they can be extracted from, as long as there are means to do so in the devices. For that reason, we briefly present three of the main domains we consider of interest for an IoT positioning system:

- Power or signal strength-based;
- Time-based;
- Space-based.

Other domains, such as natural or artificial fields, e.g., geo-magnetic field, light, sounds, or smell are out of the scope of our study, but could also serve as relevant sources of information for future IoT positioning systems.

The following subsections provide a short summary of main challenges in each of these three positioning domains and their system-wide impacts.

#### 3.1.1. Power Domain

Signal strength measurements are derived from the protocol operation, which most of the times results in a measurement of no additional cost to the device and battery consumption. However, positioning solutions in the power domain must tackle several challenges, in particular those related to the fast fluctuations of the Received Signal Strength (RSS) or of the backscattered power (BP), due to fading and shadowing caused by the surrounding environment. One key factor to model the RSS measurements relies on the possibility of understanding, with a given degree of accuracy, how the signal power changes in its surrounding environments. The signal power models as a function of the distance between the transmitter and the receiver are known as path-loss models, [10,11]. A typical empirical Log distance model is the single-slope path loss model [11]:

(1) $$P_{r}\left(d\right)=P_{r}\left(d_{0}\right)-10\eta \log_{10}\;\left(\frac{d}{d_{0}}\right)+w,$$

where $P_{r}\left(\cdot \right)<0$ is the received signal power in logarithmic scale dependent on distance *d*, $d_{0}$ is a reference distance (usually 1 m), η>0 is the path-loss exponent and $w∼log(N\left(0,\sigma^{2}\right)$) is a log-normally distributed random variable that models the slow fading phenomenon and possible RSS measurements errors (e.g., due to quantization). Both η and *w* are dependent on the propagation environment and are typically dependent on the device type and environment type. In addition, *w* can depend on factors such as device orientation and the amount of people present in the measurement area at the time of acquisition.

In terms of an IoT positioning system, the fact that one can extrapolate this information directly from the communication’s signal, which means that there is no additional cost for the device. In terms of battery, the cost will depend on the amount of positioning location requests demanded per second. Ideally, if the requirement is to have an opportunistic location, based on the sporadic communication of the device, acquiring the RSS-based positioning will have no impact on the battery life. However, if the device or the infrastructure will have to listen periodically for a specific pilot signal, acquiring the RSS-based positioning will cause further demands in terms of battery consumption. One limitation of RSS-based approaches is that some current IoT standards support only a coarse RSS measurement (e.g., in steps of 6 dB), which can adversely impact the positioning accuracy, as the noise variance $\sigma^{2}$ will increase.

**Figure 1:**
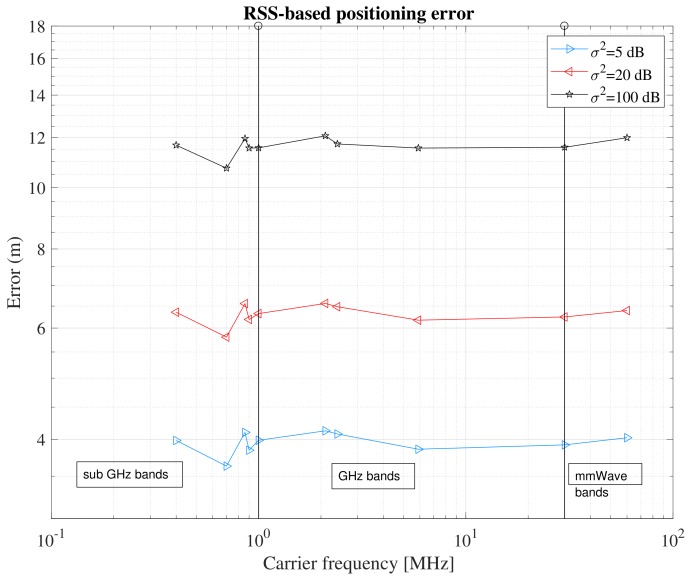
Comparative analysis of RSS-based estimates at various carrier frequencies and various AN densities.

**Figure 2:**
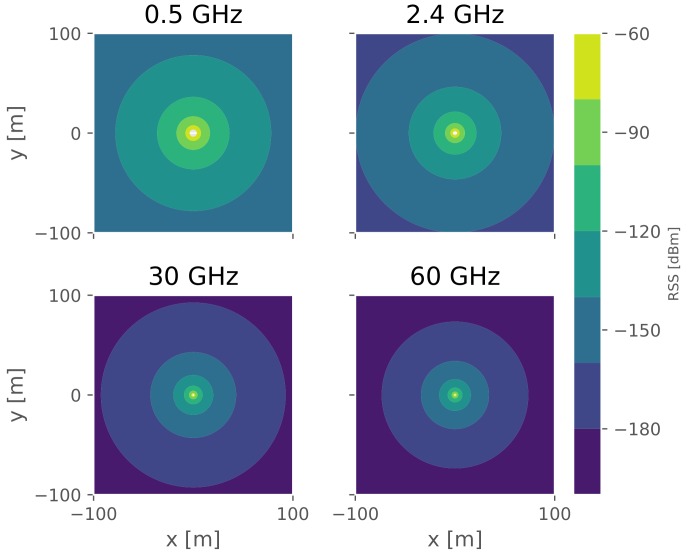
Ideal radio signal propagation at 0.5, 2.4, 30 and 60 GHz.

Another interesting aspect of the RSS measurements is that, based on simulations, RSS-based positioning errors are shown to be frequency independent (as shown later in Figure 1). However, one would expect different levels of location-based service at different frequency ranges. The frequency ranges can be coarsely divided into three categories: sub-GHz (i.e., carrier frequencies less than 1 GHz), GHz (1 to 30 GHz) and mmWave (above 30 GHz). The scattering becomes more prominent as frequency increases, thus one would expect different target positioning accuracy according to the frequency range. In addition, as the operating frequency increases, the antenna’s effective area is smaller, and the signal coverage decreases. This is possible to see in Figure 2 where the ideal signal propagation in drawn over a 100 by 100 square area, based on the Friis equation and assuming zero system gains, *G*,

(2) $$P_{r}\left(d\right)=P_{t}+G+20\log_{10}\;\left(\frac{c}{f4\pi }\right)-20\log_{10}\;\left(d\right)$$

where $P_{t}$ is the transmission power, *f* the operating frequency and c the speed of light.

Based on the signal’s behavior, it is easy to understand that a sparser infrastructure at higher frequencies will likely result in a degradation of the positioning performance (as shown later in Figure 1).

#### 3.1.2. Time Domain

Positioning estimation based on timing information is based on estimating the time-of-arrival (TOA) or the time-difference-of-arrival (TDOA) from three or more fixed access nodes and then converting those timing estimates into distances. For example, 3D location based on TOA is possible with three synchronized measurements from three known devices. The goal is to solve the following set of equations and find out the node’s location, $\xi_{n}=\left(x_{n},y_{n},z_{n}\right)$, assuming that $\xi_{a}=\left(x_{a},y_{a},z_{a}\right)$ are known coordinates:

(3) $$L_{n}=\sqrt{{\left(\xi_{a}-\xi_{n}\right)}^{2}}.$$

For TDOA, the range is now a difference of ranges, based on the TOA at the measurement device. Hence, the TDOA from a node *n* to a measurement device *m* would be written as

(4) $$L_{nm}=L_{n}-L_{m}.$$

Due to this difference, the range measurement is free of errors imposed by the measurement device’s clock, since it cancels out when subtracting the two TOA measurements.

Overall, the time measurements require synchronized clocks, either at the receiver or at the transmitter side, leading to a significant burden on device cost. This does not play well for IoT applications, which are driven by the need of having low-cost devices.

**Figure 3:**
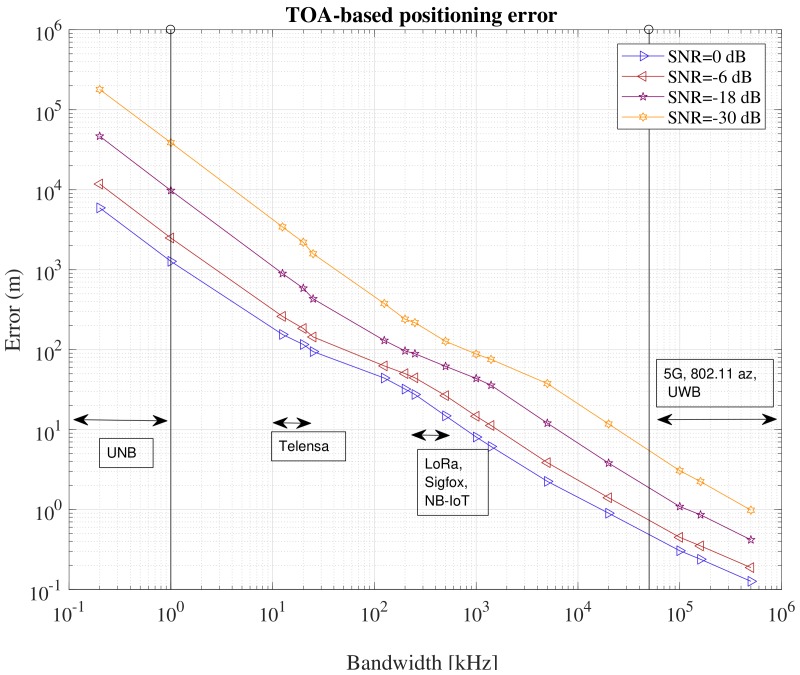
Comparative analysis of TOA-based position estimates at various bandwidths.

It is also important to keep in mind the relationship between bandwidth and accuracy for TOA measurements. This is illustrated in Figure 3, where the positioning error is plotted against the available channel bandwidth at different Signal-to-Noise Ratio (SNR) values. Clearly, sub-m positioning accuracy with time-based approaches is achievable only with high bandwidths (of the order of 100 MHz), but it is very challenging for narrowband and ultra-narrowband systems even at very high SNR.

#### 3.1.3. Space Domain

In the space domain, the ranges are estimated by measuring the angle (or direction) of arrival (AoA) for the signal of interest. Often, this is done by the means of an antenna array or a sectorized antenna. For a given device *n*, it is possible to describe its measurement at *m* as

(5) $$\left(x_{n},y_{n}\right)=r_{m}cos\left(\theta_{m}\right)+r_{m}sin\left(\theta_{m}\right),$$

where $r_{m}$ is the distance from *m* to *n* and θ the angle of arrival determined at *m*. Hence, by solving for the unknown coordinates, one can obtain a range estimate.

In summary, AoA is particularly interesting for IoT, as the major constraint for achieving angle measurements relies only on the antenna design. However, its major drawback is that the error increases with the distance to the transmitter, which means that a small deviation in the angle results in a large error for the devices at the service edge.

### 3.2. IoT Classifications

**Figure 4:**
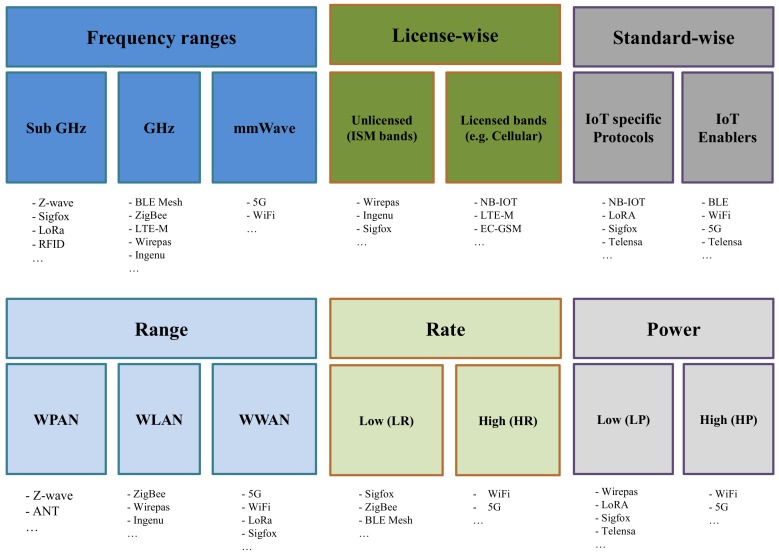
Classification of IoT networks.

While there are several domains from where to extract measurements for building knowledge of a device’s location, several limitations arise from the actual IoT system that is built upon. The goal of this subsection is to introduce the IoT technologies, by classifying them into six main categories (see Figure 4):

- *Licensed versus unlicensed*: which refers to the operation in a protected band, such as cellular bands versus operation in unlicensed bands, such as industrial, scientific and medical (ISM) bands;
- *Operating frequency bands*: which refers to the carrier frequency of each IoT technology; here, we divide the frequency spectrum into three parts: sub-GHz, GHz, and mmWave bands. Some IoT technologies spread over multiple ranges;
- *Protocols versus enablers*: which refers to whether a technology is seen as a specific IoT communication protocol or a possible wireless positioning enabler;
- *Range-based classification*: which refers to short-, medium-, or long-range operation;
- *Rate-based classification*: which refers to Low-Rate (LR) or High-Rate (HR) data rates. Typically, most IoT connectivity solutions are meant for LR high delay applications, while solutions such as WiFi and 5G cover HR and low latency applications;
- *Power-based classification*: which refers to Low-Power (LP) versus High-Power (HP) operation. Typically, LP approaches go hand in hand with LR approaches, while HP approaches go hand in hand with HP approaches. In LP operation, the devices can function for several years on batteries.

We make the following assumptions: in the range category, we consider long-range for those protocols capable of delivering more than 10 km links; for the rate category, we assume HR as those able to achieve an uplink above 10 Mbit/s and, for power consumption category, we assume as LP all those IoT systems who can support at least non-routing devices on more than two years of battery.

### 3.3. IoT System Parameters

This subsection discusses the relevant parameters for a positioning fit in an IoT system.

**Figure 5:**
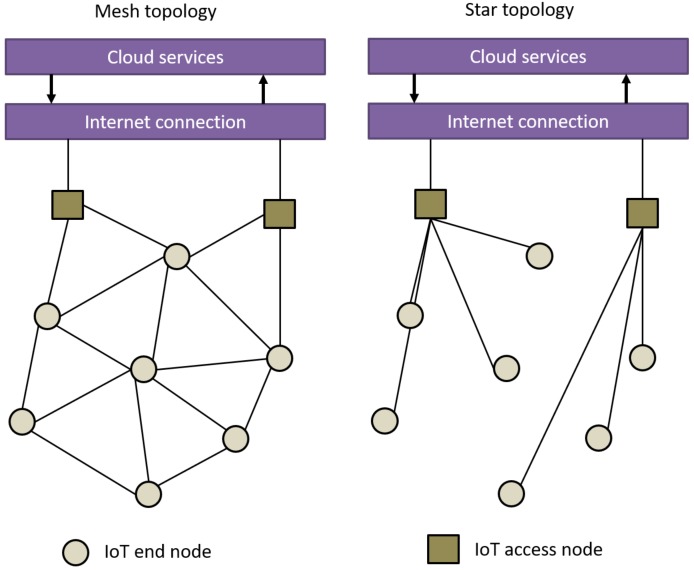
Comparison of a simplified IoT mesh and star network topology.

**Topology** relates to a message passing from one node to another and the possibility to discover new nodes in the network. The network topology, illustrated in Figure 5, has a significant impact on how nodes with known locations are discovered by others. On a mesh topology, any node can be set as a reference node, whereas, on a star topology, only the access nodes can be defined as such. The density of the fixed nodes also plays an important role in the location accuracy. For example, a denser network with a well-spread distribution of nodes is likely to provide a better location accuracy than a network with few reference nodes all placed in the same direction from the device to be located. An IoT network typically has a star or mesh topology. In a star topology, devices can only talk to their parent device, while, in a mesh topology, nodes can exchange messages between each other. Star topologies are susceptible to single points of failure, since losing the connection to the parent means that the node will be outside the network. In a mesh topology, if a link fault occurs, the device can look for any other neighbor to connect to. Thus, mesh networks provide better coverage and, implicitly, they are likely to offer better positioning accuracy than star networks;
**Range** of an IoT system is important in the sense that it defines an upper bound of the positioning error, which cannot be larger than the communication range. In this aspect, mesh-capable networks have a better footing for positioning purposes as any device can extend service without the need to have specific and dedicated infrastructure;
**Channel bandwidth** is directly related to the achievable accuracy in positioning when a TOA-based estimation is used. The Crámer–Rao lower bound for any unbiased estimator [12] of a time delay $\tau_{0}$ of a signal *S* is given as
  (6) $$\begin{array}{c} \mathrm{var}\left({\hat{\tau }}_{0}\right)\geq \frac{1}{\frac{\varepsilon }{N_{0}/2}\frac{\int_{-\infty }^{+\infty }{\left(2ßf\right)}^{2}{\left|S\left(f\right)\right|}^{2}df}{\int_{-\infty }^{+\infty }{\left|S\left(f\right)\right|}^{2}dt}}, \end{array}$$
  where ε is the signal energy, $N_{0}$ the noise spectral density and $\int_{-\infty }^{+\infty }{\left(2ßf\right)}^{2}{\left|S\left(f\right)\right|}^{2}df$ is the mean square bandwidth of the signal. However, since we have do not have all the necessary information to accurately determine each IoT signal’s spectrum density, we provide instead the multipath resolution or time-frequency resolution defined as follows:
  (7) $$\begin{array}{c} \Delta t\geq \frac{2\pi }{\Delta w}. \end{array}$$
  Equation (7) determines how the time duration Δt and the spectral bandwidth Δω relate to each other. The spectral bandwidth is defined as the bandwidth that includes most of the signal’s energy. In this study, we assume it to be equal to the channel bandwidth. Overall, what both Equations (6) and (7) show is that, for time-based approaches, it is favorable to have signals with high SNR and short time duration (i.e., higher bandwidth);
**Carrier frequency** is inversely proportional to the signal wavelength and to the path losses exhibited by the signal. As we move from sub-GHz carriers towards mmWave carriers, the path losses are stronger and stronger, which results in smaller communication ranges. The differences in path losses are due to a multitude of phenomena, but, as frequency increases, they are especially due to the smaller effective area of the devices’ antennae. Overall, combining lower carrier frequencies and mesh topologies results in an enhanced service coverage;
**Modulation types** in IoT systems rely on various digital modulation types, from Ultra Narrow Band (UNB), defined as systems with bandwidths below 1 kHz, to Ultra Wide Band (UWB) modulations, i.e., bandwidths above 500 MHz. In addition, spread spectrum (SS) or Orthogonal Frequency Division Multiplexing (OFDM) modulations are also widely encountered. The modulation type plays a big role in the achievable positioning accuracy when TOA, TDOA, or AOA methods are used, but it has little or no impact when RSS methods are used. Certain modulation-based characteristics can be exploited for positioning purposes. For example, this is the case of SS signals (e.g., LoRa, ZigBee, etc.), where the spreading pseudo-random sequence can be used to infer the signal’s travel time in a similar fashion to GNSS;
**Positioning signaling or data exchange** is the ability to use either pilot signals or sequences of data packets to provide the location of nearby devices. However, few of the existing IoT technologies support positioning-related signaling, except for most of the cellular IoT technologies (e.g., NB-IoT), which rely on the observed time difference of arrival (OTDOA), introduced in the LTE radio. Apart from the cellular IoT technologies, the future WiFi 802.11az standards also showcase a dedicated data exchange regarding the time-of-flight information to determine the location of its devices;
**Roaming** is the ability to provide continuity of service across multiple networks, owned or not by a single entity. As mobility is a keystone of most positioning applications, it is important to take note of this when looking at IoT systems. In this aspect, protocols such as Sigfox or Ingenu are at an advantage, as they operate similarly to cellular systems and they offer service across multi continents. Despite that, even proprietary solutions start to provide open application interface specifications and open guest periods in the radio access, which facilitate the exchange of data across multiple vendors and technologies;
**Network ownership** raises security and privacy concerns. Security is becoming a strong requirement in IoT systems, especially as the data access, transport and storage become more and more regulated by international and European bodies [13]. Technologies such as Ingenu and Sigfox own the entirety of the network, meaning that the transportation of data is under their full responsibility. Thus, positioning solutions enabled by such systems will be protected by the system provider, as the infrastructure device’s location will not be known to the user;
**Power consumption** is a main topic for all IoT technologies. For positioning applications, low-power consumption is crucial for the viability of several systems, especially when the goal is to continuously track and monitor inexpensive items. For example, low-power consumption is mandatory in several use cases from the logistics and construction sectors.

### 3.4. Comparing IoT Technologies and IoT Enablers

After discussing the positioning domain and the main system parameters relevant to positioning, here we present two comparative tables between 29 IoT solutions (see Table 1 and Table 2), whose goal is to sum up the key points mentioned so far and to enable an easy comparison between the different technologies. Throughout the rest of this subsection, our goal is to make comparisons and drive the reader towards a better understanding of how a certain technology would fare as the backbone of a positioning system, in a GNSS-free case.

Table 1 presents for each technology, from left to right, the network topology, network type, the impact of each measurement domain on the device battery life and cost, the achievable positioning accuracy, the most suitable domain and reported accuracy studies.

The first column maps each technology’s topology to either a star or mesh topology.

The second column presents the network type, where each entry starts with the rate, power consumption and maximum operating range offered by the technology (see section ). The operating range has a correlation with the frequency bands in Table 2.

The third column presents the impact on battery consumption and device cost for each measurement domain in discussion. While most of the technologies do not offer such capabilities, this classification assumes that it would be possible to couple the necessary measurement units to provide such information. Hence, the classification of low impact (+), medium (++) or high impact (+++) are based on what the authors expect to be the addition burden in terms of device cost and battery burden. The power domain is seen to be the one with the smallest impact, due to the fact that it would be easily available to all of these technologies.

The fourth column provides a qualitative indicator for the expected accuracy based on what the technology currently offers. When available, this information is based on the related studies.

The fifth column states the most suitable measurement domain to use with each technology. The domain is attributed based on the technology’s signal characteristics presented in Table 2 and its current capabilities.

Table 2 describes each technologies’ key physical aspects, such as frequency bands, channel bandwidth and modulation type.

Positioning services often have a high demand for power consumption. Operating a positioning based infrastructure is often tied to the need of having a fully plugged-in (powered) infrastructure. However, there are several industrial applications that would benefit from a fully battery-operated network, especially where an electricity network might not yet be present, e.g., construction sites, or for facility of service extension and maintainability.

In terms of positioning, we found that most IoT systems are yet to offer specific signaling to support accurate measurements for localization. Few of the existing IoT systems have already raised interest in the academic field in terms of their positioning capabilities, as shown in the last column of Table 1. Most of the existing studies focus on RSS-based approaches and several of them rely on low-cost probabilistic methods requiring an underlying path loss model. Few studies that focus on time-based and space-based approaches are mostly targeting the current and future cellular IoT signals, derived from LTE, such as LTE-M, which are retaining some of LTE’s positioning characteristics such as positioning-specific signaling. In addition, future 5G networks are likely to rely on time-based and space-based positioning approaches. Our paper further contributes with additional results based on RSS and time-based approaches as shown in the next sections.

In addition, we have found that network-centric positioning solutions are being favored as opposed to device-centric ones, which is often related to the limited resources at the end nodes. However, a centralized architecture places an additional burden on the network capacity and latency as the number of devices grow. For many of the IoT systems, a centralized architecture will have difficulties accommodating real-time location systems, especially due to the strict latency requirements of such systems. Integration with other high-capacity technologies, such as WiFi and 5G, could decrease the latency at the expense of per unit cost and power consumption. The support of positioning updates at very sparse intervals ought to be feasible for many IoT technologies, which will certainly find its application in several niche markets, especially if the positioning system is supported fully by battery-powered networks over a span of multiple years.

To further complement our study, we end with a perspective on what the achievable positioning accuracy is. The next two sections focus on measurement-based and simulation-based studies, respectively. We introduce simulation-based results from two systems whose performance was difficult to find as benchmarks in the existing literature, namely IEEE 802.11az and LoRa. Then, we present measurement-based results from an office environment of a positioning system built on top of the Wirepas’ mesh solution.

## 4. Simulation-Based Performance Metrics

### 4.1. Case Study 1: 802.11az IoT Enabler, Simulation-Based Results, Time Domain

In 802.11az, a position estimate is obtained by solving the hyperbolic location based on the measured TOA $t_{A_{a}}$ at the mobile side from several ANs, where *a* is the AN index, $a=1,\ldots ,N_{AN}$.

(8) $$t_{A_{a}}=t_{s}+\frac{d(Tag,AN_{a})}{c}+\sum_{a=2}^{N_{A}N}\left(\frac{d(AN_{a-1},AN_{a})}{c}+t_{f}\right),$$

where $t_{s}$ is the starting time of transmission from one AN in the network, taken arbitrarily as the first AN ($AN_{1}$), *c* is the speed of light, $d(Tag,AN_{a})$ is the geometric distance between the mobile device and the *a*-th AN, $a=1,\ldots ,N_{AN}$, $t_{f}$ is the forwarding time of the signaling message between two access nodes, and $d(AN_{a-1},AN_{a})$ is the geometric distance between the a−1-th AN and *a*-th AN. With several noisy observations of the measured time of arrivals, the IoT device can compute its position (as well as the unknown $t_{s}$). It is assumed that the AN positions and the forwarding time are known and transmitted in the signaling message. In addition to that, a minimum of four synchronized access points are needed to estimate the four unknowns $\left(x,y,z,t_{s}\right)$, with the (x,y,z) the device location.

**Figure 6:**
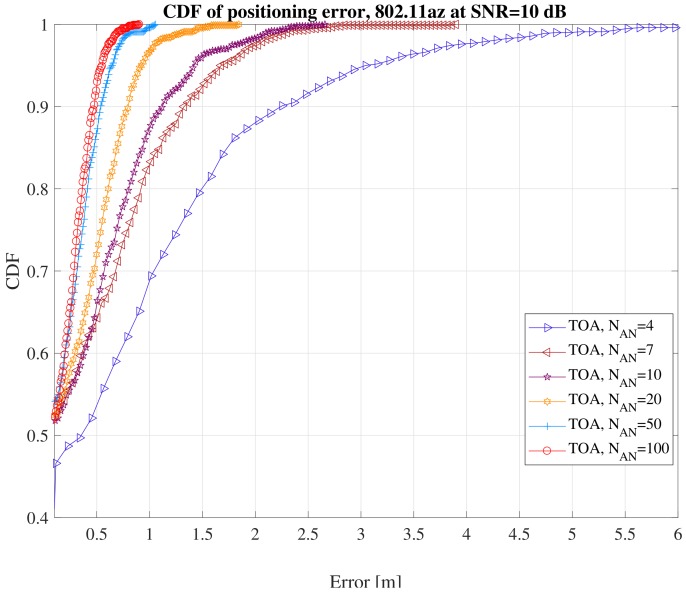
Example of 802.11az performance at various number of access nodes at signal to noise ration (SNR), SNR = 10 dB.

To understand an achievable location performance, we defined a simulation over a square area of 0.4 km^2^ at the highest bandwidth available (160 MHz). We observe in Figure 6 that this solution would be able to offer sub-meter accuracies 80% of the times when at least seven ANs are available.

### 4.2. Case-Study 2: LoRa, Simulation-Based Results, Time Domain

**Figure 7:**
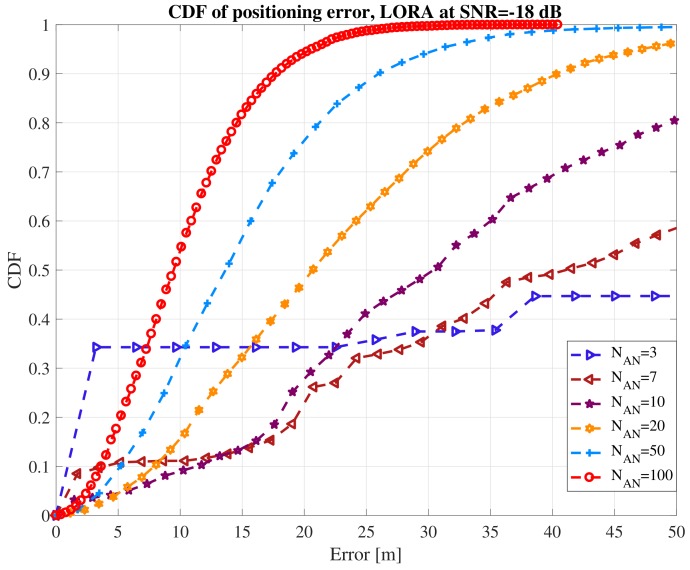
Example of LoRa performance at various numbers of access nodes.

A chirp spread spectrum (CSS) system with a 125 kHz bandwidth and a spreading factor of 7 was used in the simulations. It was assumed that we have a single-floor square indoor area of 200 m × 200 m size, in which $N_{AN}$ access nodes are distributed uniformly, with $N_{AN}$ between 3 and 100. Ten thousand Monte Carlo iterations were used to generate randomly the position of the ANs and of the IoT device. The positioning was based on TOA principle, where the TOA was estimated based on the correlation between the incoming signal and a reference CSS code. The results are shown in Figure 7 in terms of cumulative distribution function (CDF) of error, for a different number of LoRA access nodes, respectively. For three access nodes and at an SNR = −18 dB, the positioning error is higher than 50 m in more than 50% of cases. On the other hand, with 100 access nodes distributed in the 0.4 km^2^ area, we can reach below 10 m accuracy in more than 50% of cases.

## 5. Measurement-Based Performance Metrics with Wirepas IoT Platform

This section presents experimental results from an IoT positioning system built with Wirepas IoT mesh solution. The results presented in this section that were obtained are Wirepas’ offices and are based on power measurements.

**Figure 8:**
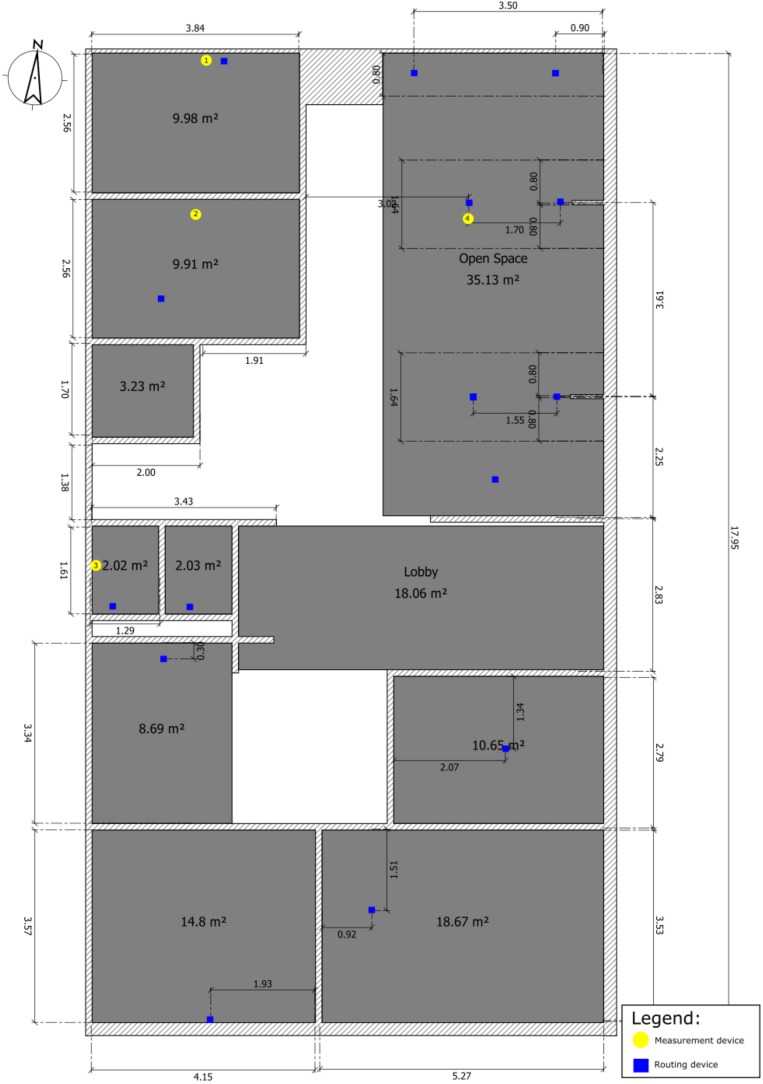
Office environment where the measurements were acquired.

The environment where measurements took place, with a total area of 180 m^2^ (10 by 18 meters), is a typical work environment with few small rooms and a large open areas (see Figure 8). Several battery powered operated devices were placed across the floor extending the network coverage in and outside the rooms. Some of these devices acted as known reference points while others as tracked devices. The reference points are identified in Figure 8 as routing devices (blue squares) and the measurement devices as yellow dots. All the devices were operating in the 2.4 GHz using Nordic’s NRF51 as the radio chipset.

In this setup, the measurement devices were statically collecting information about network beacons’ broadcast periodically by the routing devices. The information about the routing devices’ beacons, as seen by the measurement devices, was sent regularly towards the network sink. In turn, the network sink and gateway communicated the measurements to a positioning engine running on a local computer. The position engine provided a location estimate based on the known location of the routing devices and the RSS observed by the measurement devices. A location estimate was calculated by one-shot runs of a weighted centroid algorithm, meaning that no average or filtering were applied to the location estimates. However, the RSS measurements were averaged over a window of time to mitigate the channel propagation effects.

In addition to a location estimate on a global or local reference frame, the position engine also provided an area-based location. The area-based location consists of matching the location estimate to a set of geographical areas of interest (shaded areas in Figure 8). For a device to be in such area, it meant that its location estimate was found to be inside the geographic area defined by the four coordinate points of each area.

The results on Table 3 show the probability of correctly classifying the measurement devices in the areas of interest. The percentage is calculated by summing the amount of location estimates in the node’s correct area versus the total amount of location estimates in any other area of interest.

The results on Table 3 show that, during a day, the devices were correctly located inside the logical area where they were known to be at more than 90% of the time.

## 6. Conclusions

We believe positioning is important not only for IoT end applications, but also to support network self-management. Our paper addresses the lack of comprehensive studies comparing IoT solutions and their fit-for-positioning applications. The paper first covered three possible measurement domains from which IoT devices could derive their location. Afterwards, we focused on classification of the IoT solutions and we discussed several system parameters that should be considered when designing a positioning system. We concluded our study with a comparative table and discussion between multiple IoT and other wireless solutions. We also provided an overview of achievable system performance with unique results for three positioning systems built on top of IEEE 802.11az, LoRa and Wirepas, respectively.

Overall, based on our study, we conclude that power-domain positioning currently offers the best trade-off between implementation cost and positioning accuracy for low-power systems. Dedicated positioning signaling as well as space-based approaches are some of the feasible ways to push for higher accuracy and still offer low-power operation. Cooperation with other wireless technologies, such as WiFi and 5G, could allow for mobility support and ability to operate at large scales when low-power operation is not critical.

## Figures and Tables

**Table 1:** Summary of key positioning related aspects for several IoT protocols and IEEE 802.11∗ family protocols.

Impact on (Battery, Device Cost) per Domain ^1^								
Technology	Network Topology	Network Type	Time-Based Positioning	Power-Based Positioning	Space-Based Positioning	Achievable Positioning Accuracy ^2^	Most Suitable Domain	Accuracy Studies
5G	star	HR/HP-Short range	+, +	+,+	+,+	High	Time	[14,15]
ANT+	mesh	LR/LP-Short range	+,+++	+,+	++,++	Low	Power	
BLEmesh	mesh	LR/LP-Short range	+,+++	+,+	++,+	Medium	Power	[11,16,17]
Dash7	star	LR/LP-Long range	+,+++	+,+	++,++	Low	Power or Space	
EC-GSM-IOT	star	HR/LP-Long range	+,+++	+,+	++,++	Low	Power	
EnOcean	mesh	LR/LP-Long range	+,+++	+,+	++,++	Low	Power or Space	
Ingenu /RPMA	star	LR/LP-Long range	+,+++	+,+	++,++	Medium	Power or Space	
ISA101.11a	mesh	LR/LP-Short range	+,+++	+,+	++,++	Medium	Power or Space	
LoRa	star	LR/LP-Long range	+,++	+,+	++,++	Medium	Power	[18]
LTE-M	star	LR/LP-Long range	+,+	+,+	++,++	Medium	Time	[2]
MiWi	mesh	LR/LP-Long range	+,+++	+,+	++,++	Medium	Power	
NB-IoT	star	LR/LP-Long range	+,+	+,+	++,++	Medium	Time	[2]
RFID	star	LR/LP-Short range	+,+++	+,+	++,++	Medium	Power	[19,20,21,22,23]
Sigfox	star	LR/LP-Long range	+,+++	+,+	++,++	Medium	Power	[24,25]
Telensa	star	LR/LP-Long range	+,+++	+,+	++,++	Low	Power or Space	
Thread	mesh	LR/LP-Short range	+,+++	+,+	++,++	Medium	Power	
Weightless-N	star	LR/LP-Long range	+,+++	+,+	++,++	Medium	Power or Space	
Weightless-P	star	LR/LP-Long range	+,+++	+,+	++,++	Low	Power or Space	
Weightless-W	star	LR/LP-Long range	+,+++	+,+	++,++	Medium	Power	
WirelessHART	mesh	LR/LP-Short range	+,+++	+,+	++,++	Medium	Power	
WiFi802.11af	star	HR/HP-Long range	+,+	+,+	++,++	High	Time	
WiFi802.11ah/HaLoW	star	LR/LP-Long range	+,+	+,+	++,++	High	Time	
WiFi802.11az	star	HR/HP-Short range	+,+	+,+	++,++	High	Time	
WiFi802.11p (V2X)	mesh	HR/HP-Short range	+,+	+,+	++,++	High	Time	[26,27]
Wirepas	mesh	HR-Long range	+,+++	+,+	++,++	Medium	Power	
WiSUN	mesh	LR/LP-Long range	+,+++	+,+	++,++	Medium	Power	
ZigBee/ZigBee-NaN	mesh	LR/LP-Long range	+,+++	+,+	++,++	Medium	Power	[28,29,30]
Z-Wave	mesh	LR/LP-Long range	+,+++	+,+	++,++	Medium	Power or Space	

^1^ (+, +): low impact, (++, ++): medium, (+++, +++): high impact; ^2^ assuming implementation without external sensors, such as GNSS.

**Table 2:** Summary of key physical layer parameters for several IoT protocols.

Technology	Frequency Bands	Channel Bandwidth (MHz)	Modulation Type (UNB/NB/ SS/OFDM/UWB)
**5G**	GHz, mmWave	<100	OFDM
**ANT+**	GHz	1	NB
**BLE mesh**	GHz	1	NB
**Dash7**	sub-GHz	0.025, 0.200	NB
**EC-GSM-IOT**	sub-GHz	0.2	NB
**EnOcean**	sub-GHz	0.0625	NB
**Ingenu**	sub-GHz and GHz	1	SS
**ISA101.11a**	GHz	5	SS
**LoRa**	sub-GHz	0.125, 0.500	SS
**LTE-M**	sub-GHz and GHz	1.08, 1.4	OFDM
**MiWi**	sub-GHz and GHz	0.040, 0.250	NB
**NB-IoT**	sub-GHz and GHz	0.18	NB, OFDM
**RFID**	sub-GHz and GHz	0.2	NB
**Sigfox**	sub-GHz	0.2	UNB
**Telensa**	sub-GHz	0.1	NB
**Thread**	GHz	5	NB
**Weightless-N**	sub-GHz	0.2	UNB
**Weightless-P**	sub-GHz	0.0125	NB
**Weightless-W**	sub-GHz	5	SS
**WirelessHART**	GHz	0.25	SS
**WiFi802.11af**	sub-GHz	8	OFDM
**WiFi802.11ah**	sub-GHz	1, 2, 4, 8, 16	OFDM
**WiFi802.11az**	GHz, mmWave	20, 40, 60, 80, 160	OFDM
**WiFi802.11p (V2X)**	GHz	10	OFDM
**Wirepas**	sub-GHz and GHz	0.126, 0.5	NB
**WiSUN**	sub-GHz and GHz	0.2–1.2	NB, SS and OFDM
**ZigBee**	sub-GHz and GHz	0.6, 1.2, 2	SS
**ZigBee-NaN**	sub-GHz	0.6, 1.2, 2	SS
**Z-Wave**	sub-GHz	0.2	NB

**Table 3:** Experimental results with an IoT testbed using Wirepas connectivity with 60 fixes per second and static nodes.

Area (m^2^)	Office Hours	Outside Office Hours	All Day
	% of Correct Location Area Classification		
10	95.41	89.47	91.16
10	96.16	97.59	97.18
2	91.56	94.85	93.90
3	96.76	99.20	98.50
Mean	95.51	95.61	95.57
